# Multi-drug-resistant organisms are the main pathogens of surgical-site infection after colorectal surgery: a retrospective study

**DOI:** 10.1016/j.infpip.2026.100510

**Published:** 2026-01-16

**Authors:** Xuexia Yang, Yinghui Xiong, Qianqian Ye, Juan Hu, Duoduo Li, Zhenguo Liu, Pengcheng Zhou

**Affiliations:** a Department of Infectious Diseases, The Third Xiangya Hospital, Central South University, China; b Hunan Key Laboratory of Viral Hepatitis and Department of Infectious Diseases, Xiangya Hospital, Central South University, China; c Department of Pharmacy, Xiangya Hospital, Central South University, China

**Keywords:** Colorectal surgery, Surgical-site infections, Multiple-drug-resistant organism, Risk factors

## Abstract

**Objective:**

This study aimed to investigate the incidence, disease burden and risk factors of surgical-site infections (SSIs) after colorectal surgery, particularly caused by multi-drug-resistant organism (MDRO) infections, to provide evidence for the control and prevention of SSIs after colorectal surgery.

**Methods:**

This retrospective study included patients who underwent colorectal surgery at Xiangya Hospital, Central South University, between 2020 and 2021. Univariate and multi-variate analyses were performed to identify risk factors.

**Results:**

In total, 2297 patients who had undergone colorectal surgery were included, of which 94 experienced SSIs following surgery. Of the 94 patients, 54 had specific pathogens identified. Furthermore, of these 54 patients, 44 were infected with an MDRO. The main pathogen causing SSIs was *Escherichia coli*, which was isolated from 43 patients. *E. coli* isolates showed high levels of resistance to cefazolin and cefuroxime but low resistance to imipenem, piperacillin and tazobactam. Low albumin levels before surgery, prior use of antibiotics, pre-operative intensive care unit (ICU) admission, mechanical ventilation, central venous indwelling catheter and blood transfusion were statistically significant risk factors for SSIs. Prior use of antibiotics and blood transfusion were independent risk factors for SSIs. Pre-operative ICU admission, mechanical ventilation and high pre-operative venous blood glucose levels were statistically significant risk factors for MDRO infection, and pre-operative ICU admission was identified as the only independent risk factor.

**Conclusion:**

MDROs were the most common pathogens of SSIs after colorectal surgery. Prior antibiotic use, blood transfusion and pre-operative ICU admission are key independent risk factors for SSIs and MDRO infections.

## Background

Surgical-site infections (SSIs) are the most common reason for health-care-associated infections in patients who undergo colorectal surgery [1]. In recent years, the use of invasive procedures and broad-spectrum antibiotics has increased [2], resulting in the emergence of multi-drug-resistant organisms (MDROs), which pose a serious threat to the prevention and treatment of SSIs [3]. MDROs exhibit non-susceptibility to at least one agent in three or more antimicrobial categories typically used for empirical or targeted therapy. The causes and risk factors of SSIs caused by MDROs have not been well explained. As a result, effectively selecting antibiotics in the era of MDROs is challenging.

## Objects and methods

This study was approved by the Ethics Committee of Xiangya Hospital, Central South University. Patients who had undergone colorectal surgery between 1^st^ January, 2020 and 31^st^ December, 2021 at Xiangya Hospital, Central South University, were included in the study. The diagnostic criteria for SSIs were based on the nosocomial infection diagnostic criteria (trial) of the Ministry of Health of China [4]. Patients with SSIs after colorectal surgery were included in the infection group. The infection group exclusion criteria were as follows: those with postoperative infections at other sites, those with existing intestinal perforation and intestinal obstruction before admission, those accepting other sites of surgery concurrently and those unable to provide complete data. Patients who had undergone colorectal surgery at the same time were included in the control group. In the infection group, patients were divided into two subgroups: the MDRO group and the non-multi-drug-resistant organism (NMDRO) group. Statistical analysis was performed using the SPSS 25.0 software(International Business Machines Corporation [IBM]). Chi-squared test, *t*-test and Wilcoxon rank-sum test were used for data analysis based on the characteristics of the data. Risk factors were analysed using a logistic regression analysis. Due to the presence of complete separation in the data for several predictor variables, which resulted in unstable maximum likelihood estimates with extreme odds ratios, the final multi-variate analysis was performed using Firth’s penalised logistic regression. This method provides bias-reduced estimates that are particularly suitable for datasets with separation or rare events. A *P* value <0.05 was considered statistically significant.

## Results

### Incidence and disease burden of SSIs

Of the 2297 patients included in the study, 94 (4.09%) had SSIs; among them, 18 had superficial incision infection, 12 had deep incision infection and 64 had organ compartment infection. Of these 94 patients, 44 had an MDRO infection and 50 had an NMDRO infection. The infection group had a heavier disease burden than the control group (Table I).

**Table I:** Disease burden

Disease burden	Infection (*N* = 94)	Control (*N* = 93)	*P*	MDRO (*N* = 44)	NMDRO (*N* = 50)	*P*
Hospitalisation costs (RMB)	85,716.96 (72,069.25–106707.84)	56,318.06 (49,657.60–64092.41)	<0.001	92,498.39 (81,907.88–115979.64)	80,678.06 (68,591.42–101245.72)	0.014
Length of stay (day)	27.50 (22.00–34.00)	15.00 (12.00–18.00)	<0.001	25.00 (21.00–32.00)	29.00 (22.00–36.00)	0.153

MDRO = multi-drug-resistant organism; NMDRO = non-multi-drug-resistant organism.

### Clinical characteristics

Clinical characteristics of patients are listed in Table II. The albumin concentration in the control group was higher than that in the infection group.

**Table II:** Clinical characteristics of patients

	Infection (*N* = 94)	Control (*N* = 93)	*P*
Age	59.69 ± 10.18	60.00 ± 9.45	0.830
Gender (male/female)	68/26	67/26	0.964
Operation time (min)	225 (80.00–455.00)	210 (108.00–380.00)	0.084
Pre-operative serum creatinine (mmol/L)	75.50 (65.00–89.00)	78.00 (68.75–86.55)	0.428
Pre-operative total bilirubin (mmol/L)	9.25 (6.78–14.75)	10.40 (7.40–14.20)	0.771
Pre-operative albumin level (g/L)	37.35 (34.58–40.20)	38.50 (36.50–40.40)	0.034
Pre-operative fasting blood glucose (mmol/L)	5.19 (4.77–5.86)	4.97 (4.46–5.63)	0.093

### Pathogens distribution

The primary pathogens detected were *Escherichia coli* (*N* = 43, 57.33%), *Pseudomonas aeruginosa* (*N* = 10, 13.33%), *Enterococcus avium* (*N* = 7, 9.33%), *Klebsiella pneumoniae* (*N* = 5, 6.67%), *Enterococcus faecalis* (*N* = 5, 6.67%), *Enterococcus faecium* (*N* = 1, 1.33%) and other strains (*N* = 4, 5.34%) in 54 patients who had unambiguous etiological examination results. *E. coli* and *K. pneumoniae* had high resistance rates to ampicillin, cefazolin, cefuroxime, ceftriaxone, levofloxacin, ciprofloxacin and gentamicin. The detailed information of susceptibility results of pathogen is showed in the Supplementary material. It should be specifically noted that the percentages of all subsequent pathogens (e.g., 57.33%) are calculated based on the total number of strains isolated from the patients.

### Risk factors for SSIs and MDRO infection

The univariate logistic regression analysis revealed that low albumin levels before surgery, prior use of antibiotics, pre-operative ICU admission, mechanical ventilation, central venous indwelling catheter and blood transfusion were statistically significant risk factors for SSIs. The Firth’s penalised logistic regression revealed that prior use of antibiotics and blood transfusion were independent risk factors for SSIs (Table III). The univariate analysis revealed that pre-operative ICU admission, mechanical ventilation and high pre-operative venous blood glucose levels were statistically significantly different between the MDRO group and the NMDRO group. Pre-operative ICU admission was identified as the only independent risk factor for MDRO in SSIs (Table IV).

**Table III:** Risk factors for surgical-site infections

Factors	Infection (*N* = 94)	Control (*N* = 93)	Univariate analysis	Multi-factor logistic regression		
			*P*	*P*	OR	95% CI
Albumin before surgery (g/L)	37.35	38.50	0.034	0.237	0.95	0.87–1.03
Prior use of antibiotics	16/94	0/93	<0.001	<0.001	36.87	4.75–4748.68
Pre-operative ICU admission	7/94	0/93	0.014	0.098	7.90	0.72–1058.30
Mechanical ventilation	14/94	1/93	0.001	0.125	3.82	0.70–38.11
Central venous indwelling catheter	87/94	75/93	0.017	0.179	1.84	0.76–4.88
Blood transfusion	8/94	0/93	0.007	0.042	10.46	1.07–1396.25

CI = confidence interval; ICU = intensive care unit; OR = odds ratio.

**Table IV:** Risk factors for multi-drug-resistant organism infection

Factors	MDRO (*N* = 44)	NMDRO (*N* = 50)	Univariate analysis	Multi-factor logistic regression		
			*P*	*P*	OR	95% CI
Pre-operative ICU admission	7/44	0/50	0.004	0.032	6.26	1.16–63.63
Mechanical ventilation	11/44	3/50	0.018	0.861	0.82	0.07–6.72
High pre-operative venous blood glucose	7/44	6/50	0.042	0.578	1.41	0.42–4.78

CI = confidence interval; ICU = intensive care unit; MDRO = multi-drug-resistant organism; NMDRO = non-multi-drug-resistant organism; OR = odds ratio.

## Discussion

SSIs are the most frequent postoperative issue of colorectal surgery and have a serious negative impact on patients’ prognoses and significantly increase the social and financial burden. In this study, we conducted a thorough investigation of the incidence rate, disease burden, aetiology and risk factors for SSIs following colorectal surgery. A high rate of SSIs (up to 4.09%) was detected. Moreover, a significantly longer overall hospital stay and consequently higher hospital costs were detected in the infection group than in the control group.

We observed that MDROs were the most significant pathogens at the surgery site. Pre-operative prophylactic antibiotics for abdominal surgery include cefazolin, cefoxitin, cefuroxime and ceftriaxone, but MDROs have a low susceptibility to them. Despite the implementation of strict antibiotic management policies and infection control measures in hospitals, low sensitivity to cephalosporin may still be observed. This can be attributed to several factors: spread of resistance genes, antibiotics’ selective pressure, complex clinical situations, diversity of infection sources and ambiguity in antibiotic indications. For patients who were accurately identified as high risk for SSIs, further research is necessary to determine whether additional antibiotics with high resistance barriers should be administered for peri-operative prophylaxis, such as piperacillin, tazobactam or ertapenem.

We have identified some factors that predict SSIs. Albumin is the most popular index used to measure patients’ nutrition. Low albumin can lower patients’ immunity and delay the healing of surgical incisions, thereby negatively impacting patients’ postoperative prognoses. This study also identifies blood transfusions as a risk factor, which is consistent with the findings of Ejaz *et al.* [5]. SSIs may be associated with the immunosuppressive impact of transfusion-related immune modulation. Risk factors for SSIs include mechanical ventilation and central venous indwelling catheters. As invasive procedures, these procedures greatly increase the risk of health-care-acquired infections. However, prior use of antibiotics and blood transfusion were identified as the independent risk factors in the multi-factor logistic regression analysis. Prior antibiotic exposure can disrupt protective microbiota and select for resistant pathogens, while blood transfusion may induce immunomodulatory effects. Furthermore, we found that pre-operative ICU admission and high pre-operative venous blood glucose levels were risk factors for SSIs caused by MDROs. Moreover, pre-operative ICU admission was identified as a risk factor for both SSIs and infection with MDROs. ICU staff and the environment may have colonisation with MDROs, making ICU a location with a higher incidence of MDRO infection. Surgical patients are susceptible to acquiring MDROs during or after admission to the ICU, and the acquired MDROs may not susceptible to peri-operative prophylactic antibiotics, thereby increasing the risk of infection. Diabetes is a recognised independent risk factor for SSIs [6]. Diabetes impairs immunity, and the incision exudate’s high-glucose environment encourages bacterial growth, thereby increasing the risk of SSIs.

This study has several limitations. First, since this was a single-centre, retrospective study, all data were collected from patients’ daily progress notes and follow-up notes, influencing the quality of the data used. Despite this, the study’s low rate of missing data gives us a reason for confidence. However, the single-centre nature of the study limits generalisability of these results to different healthcare institutions. Second, since this was a single-centre study and it excluded patients with other infections (to minimise the impact of confounding effects on the primary outcome), this may have limited the generalisability of the results. Therefore, the conclusions drawn from this study apply to populations without co-infections and it is suggested that future studies expand their population range.

In conclusion, SSIs caused by MDROs following colorectal surgery are a crucial problem. Prior antibiotic use, blood transfusion and pre-operative ICU admission are key independent risk factors for SSIs and MDRO infections. Prophylactic use of antibiotics with high-resistance barriers for patients at a high risk of SSIs may be necessary.

## CRediT authorship contribution statement

**X. Yang:** Writing – original draft, Methodology. **Y. Xiong:** Methodology. **Q. Ye:** Data curation. **J. Hu:** Methodology. **D. Li:** Data curation. **Z. Liu:** Methodology. **P. Zhou:** Conceptualization.

## Consent for publication

Written informed consent to publish results was obtained from all the interviewees.

## Data availability

Data for this study may be made available upon request from the corresponding author.

## Ethics statement

This study is authorised by the Ethics Committee of Xiangya Hospital, Central South University.

## Disclosure

This paper has been uploaded to Research Square as a preprint: https://www.researchsquare.com/article/rs-2488543/v1.

## Funding sources

This work was supported by the Chinese Preventive Medicine Association hospital infection discipline development of young talent lift project support (grant number: CPMA-HAIC-20240129001).

## Conflict of interest statement

The authors declare that they have no competing interests.
