# Supplementary material for: Multi-drug-resistant organisms are the main pathogens of surgical-site infection after colorectal surgery: a retrospective study
Source: Infect Prev Pract. 2026 Jan 16;8(2):100510. doi: 10.1016/j.infpip.2026.100510 (PMC12972696; doi:10.1016/j.infpip.2026.100510)
Supplement: Multimedia component 1 [file mmc1.docx]

Supplement Material:

**1. Susceptibility results of a pathogen in SSIs**

**1.1 Drug resistance rate of Gram-negative bacteria to** **SSIs after colorectal surgery**

In contrast to imipenem and piperacillin tazobactam, *Escherichia coli* had high resistance to ampicillin, cefazolin, cefuroxime, ceftriaxone, levofloxacin, ciprofloxacin, gentamicin, and ampicillin sulbactam. *Klebsiella pneumonia* had a high level of resistance to Ampicillin, cefazolin, cefoxitin, cefuroxime, and ceftriaxone, while had a low level resistance to imipenem, levofloxacin, ciprofloxacin, gentamicin, and piperacillin tazobactam. *Pseudomonas aeruginosa* were only moderately resistant to all antimicrobials. Table 1 shows this specific information in detail.

**Table 1. Susceptibility of Gram-negative bacteria to antimicrobials**

| Antimicrobials | ***Escherichia coli*** **(n=43)** | | ***Klebsiella pneumoniae*** **(n=5)** | | ***Pseudomonas aeruginosa*** **(n=10)** | |
| --- | --- | --- | --- | --- | --- | --- |
|  | R (%) | S (%) | R (%) | S (%) | R (%) | S (%) |
| Ampicillin | 90.70 | 4.65 | 100.00 | 0 | / | / |
| Cefazolin | 72.09 | 18.60 | 60.00 | 40.00 | / | / |
| Cefoxitin | 25.58 | 53.49 | 40.00 | 60.00 | / | / |
| Cefuroxime | 67.44 | 13.95 | 60.00 | 40.00 | / | / |
| Ceftriaxone | 62.79 | 23.26 | 40.00 | 60.00 | / | / |
| Imipenem | 2.33 | 97.67 | 0 | 100.00 | 0 | 70.00 |
| Levofloxacin | 46.51 | 32.56 | 0 | 100.00 | 10.00 | 70.00 |
| Ciprofloxacin | 51.16 | 23.26 | 0 | 100.00 | 0 | 70.00 |
| Gentamicin | 41.86 | 44.19 | 0 | 100.00 | 0 | 80.00 |
| Ampicillin sulbactam | 44.19 | 44.19 | 20.00 | 80.00 | 10.00 | 60.00 |
| Piperacillin tazobactam | 6.98 | 79.07 | 0 | 80.00 | 10.00 | 60.00 |

**1.2 Drug resistance rate of Gram-positive bacteria to SSIs after colorectal surgery**

*Enterococcus faecalis* were only moderately resistant to all antimicrobials. A high level of resistance to ampicillin, levofloxacin, ciprofloxacin, and tetracycline was present in *Enterococcus faecium*, but not to gentamicin, tigecycline, and vancomycin. A high level of gentamicin and tetracycline resistance was present in *Enterococcus avium*, whereas ampicillin, levofloxacin, ciprofloxacin, tigecycline, and vancomycin resistance were absent. Table 2 displays this comprehensive information.

**Table 2. Susceptibility of Gram-positive bacteria to antimicrobials**

| Antimicrobials | ***Enterococcus faecalis (n=5)*** | | ***Enterococcus faecium (n=1)*** | | ***Enterococcus avium (n=7)*** | |
| --- | --- | --- | --- | --- | --- | --- |
|  | R (%) | S (%) | R (%) | S (%) | R (%) | S (%) |
| Ampicillin | 0 | 100.00 | 100.00 | 0 | 28.57 | 71.43 |
| Levofloxacin | 0 | 80.00 | 100.00 | 0 | 14.29 | 85.71 |
| Ciprofloxacin | 0 | 40.00 | 100.00 | 0 | 14.29 | 85.71 |
| Gentamicin | 20.00 | 80.00 | 0 | 100.00 | 71.43 | 28.57 |
| Tigecycline | 20.00 | 80.00 | 0 | 100.00 | 0 | 100.00 |
| Tetracycline | 40.00 | 60.00 | 100.00 | 0 | 100.00 | 0 |
| Vancomycin | 0 | 100.00 | 0 | 100.00 | 0 | 100.00 |
